# Supplementary material for: Content-rich biological network constructed by mining PubMed abstracts
Source: BMC Bioinformatics. 2004 Oct 8;5:147. doi: 10.1186/1471-2105-5-147 (PMC528731; doi:10.1186/1471-2105-5-147)
Supplement: Additional File 2 — The original results of the above study (non-essential files are deleted to keep the file size under the limit set by BMC bioinformatics). [file 1471-2105-5-147-S2.bz2 › chilibotAdditionalFile2/dip20/21ID9129204E79/html/TNFRSF1A_TRAF2.html]

 


 **TNFRSF1A** and **TRAF2** 
  
Found 140 abstracts in PubMed, retrieved 20.  
 

 What does Google say? 
 PDF only 
| .edu only 

---

**Interactive relationship** (e.g. stimulation, inhibition, etc)

**Neutral relationship**- Apoptotic crosstalk of TNF receptors TNF R2 induces depletion of  **TRAF2**  and IAP proteins and accelerates TNF R1  [ **TNFRSF1A** ]  dependent activation of caspase 8.  Ref: 12077366 J Cell Sci, 2002
- In an in vitro binding assay, the intracellular domain of TNF R1  [ **TNFRSF1A** ]  bound TRADD, RIP and  **TRAF2**  but did not bind FADD or caspase 8.  Ref: 12721308 J Biol Chem< MedlineTA>J Biol Chem, 2002
- TNFR  [ **TNFRSF1A** ]  associated factor 2  **TRAF2**  is an adapter protein that links several members of the TNFR  [ **TNFRSF1A** ]  family to downstream signaling pathways.  Ref: 12218092 J Immunol, 2002
- Life cell imaging and confocal microscopy revealed that both TNF R1  [ **TNFRSF1A** ]  and TNF R2 recruited the anti apoptotic factor cIAP1 in a  **TRAF2**  dependent manner.  Ref: 12077366 J Cell Sci, 2002
- Thus, TNF R2 may compete with TNF R1  [ **TNFRSF1A** ]  for the recruitment of newly synthesized  **TRAF2**  bound anti apoptotic factors, thereby promoting the formation of a caspase 8 activating TNF R1  [ **TNFRSF1A** ]  complex.  Ref: 12077366 J Cell Sci, 2002
- The TNF induced NF kappaB activation cascade involving TNFR1  [ **TNFRSF1A** ]  TNF receptor associated death domain TNF receptor associated factor 2  [ **TRAF2** ]   **TRAF2**  NF kappaB inducing kinase NIK IkappaBalpha kinase was interrupted at the  **TRAF2**  and NIK sites by calagualine, which would account for its suppression of NF kappaB reporter gene expression.  Ref: 12565172 Cancer Lett, 2003

**Non-interactive relationship** (e.g. studied together, co-existance, homology, etc.)

- The C terminal 16 amino acid residues of TNFR2 are critical for Etk association and activation, and this Etk binding and activating motif in TNFR2 is not overlapped with the TNFR  [ **TNFRSF1A** ]  associated factor type 2  **TRAF2**  binding sequence.  Ref: 12370298 Mol Cell Biol, 2002
- When TNFalpha bound TNFR2 interacts with TNFR  [ **TNFRSF1A** ]  associated factor 2  **TRAF2** , expression of survival antiapoptotic genes is up regulated.  Ref: 12533416 Biol Reprod, 2003
- The initial plasma membrane bound complex complex I consists of TNFR1  [ **TNFRSF1A** ] , the adaptor TRADD, the kinase RIP1, and  **TRAF2**  and rapidly signals activation of NF kappaB.  Ref: 12887920 Cell, 2003
- Here we show that upon TNFalpha binding, TNFR1  [ **TNFRSF1A** ]  translocates to cholesterol and sphingolipid enriched membrane microdomains, termed lipid rafts, where it associates with the Ser Thr kinase RIP and the adaptor proteins TRADD and  **TRAF2** , forming a signaling complex.  Ref: 12753742 Immunity, 2003
- Adenosine also suppressed NF kappaB dependent reporter gene expression activated by TNF or by overexpression of TNFR1  [ **TNFRSF1A** ] , TRAF 2  [ **TRAF2** ] , NIK, and p65 subunit of NF kappaB.  Ref: 12606947 Oncogene, 2003
- At the same time, NF kB can be activated by TNF alpha and IL 1, which would then reach TNFR1  [ **TNFRSF1A** ] , TRADD,  **TRAF2** , MEK, JNK and c Jun activation or return to the previous IkB route.  Ref: 12542760 Tissue Antigens< MedlineTA>Tissue Antigens, 2003
- TCR independent CD30 mediated IL 13 release correlated with activation of c Jun N terminal kinase, p38 mitogen activated protein kinase MAPK, and NF kappaB, and was completely inhibited by the expression of a TNFR  [ **TNFRSF1A** ]  associated factor 2  **TRAF2**  dominant negative transgene  **TRAF2** .DN Tg, but not by that of an I kappaBalpha dominant negative transgene.  Ref: 12193714 J Immunol, 2002
- The constitutive expression of mRNA for TNF receptors TNFR  [ **TNFRSF1A** ] , including TNFRI and TNFRII, and the adapter molecules, such as the TNF receptor associated death domain protein TRADD, Fas associated death domain protein FADD, receptor interacting protein RIP and TNF receptor associated factor 2  [ **TRAF2** ]  TRAF 2  [ **TRAF2** ]  were analyzed by reverse transcriptase RT PCR in bone marrow samples from control, MDS and AML cases.  Ref: 12681357 Leuk Res, 2003
- The induction of IkappaBalpha degradation by DNA damage was normal in TNF R1  [ **TNFRSF1A** ] ,  **TRAF2** , TRAF5 and FADD fibroblasts or when de novo protein synthesis was blocked.  Ref: 12654725 Genes Dev, 2003
